# The gender affirming surgery in a conservative religious country: the Lebanese experience

**DOI:** 10.2144/fsoa-2022-0039

**Published:** 2023-04-11

**Authors:** Wael Abdallah, Bernard Najib, Khalil Khalil, David Atallah

**Affiliations:** 1 Department of Gynecology & Obstetrics, Hôtel-Dieu de France University Hospital, Saint Joseph University, Beirut, Lebanon

**Keywords:** ethics, gender affirming surgery, law, Lebanon, religion

Lebanon is considered liberal and democratic in comparison with Arab countries; but when it comes to transgender people, many legal and social challenges are present. In the penal code, article 534 identifies *“Any sexual intercourse contrary to the order of nature is punished by imprisonment for up to 1 year”* [1,2]. In Lebanon, transgender people are frequently victims of a transphobic culture, which originates from a lack of understanding in society. They are often erroneously conflated with the gay community, who are also victims of systemic prejudice [3] . As medical doctors, we find ourselves responsible for providing medical care to this community despite all the social, legal and religious challenges, based on ethical principles. Of concern, we are writing this letter to share our experience with transgender patients since 2004 in our Obstetrics and Gynecology department in a tertiary referral hospital in Beirut, Hotel Dieu de France.

The start of our experience was tragic. In 2004, a trans woman who underwent successful gender affirming surgery in our clinic was killed by her children. This crime was enough to lead us to reconsider the strategies adopted in our institution to provide a plan of care for transgender patients.

Multiple ethical problems are assumed in the face of every transgender patient:

-  Autonomy: While every patient must have the right to present their self-identification in the settings of name and gender, we find ourselves obliged to insert them in the medical record upon their identity card, as requested legally and by medical insurance. However, changing your gender identity was not legally possible in Lebanon. The Court of Appeals in Beirut confirmed in 2015 for the first time the right of a transgender man to change his legal gender identity, granting him access to necessary treatment and reserving his privacy [4]. This is the only judgement so far in Lebanon of a legally recognized gender identity change. This legal revolution gave hope to transgender people to have the right to change their gender identity.
-  Nonmaleficence: it is a principle that pushes the provider to minimize harm to patients. The assessment of the complaint is necessary to identify which treatment would be the most beneficial for each patient. Although our department can offer surgical treatment for these patients, we are still limited in Lebanon by the number of competent surgeons able to provide these operations. A multidisciplinary staff was created, based on social and psychological assistants, endocrinologists, surgeons and an ethical committee before providing any treatment for the patient. The principle of confidentiality is critically essential within the therapeutic setting to keep the trust between providers and patients.
-  Justice: we cannot apply this principle to the majority of cases. This principle should ensure equal distribution of healthcare resources between patients. Unfortunately, all the medical insurances do not cover sexually transmitted diseases, hormonal therapy and gender affirming surgeries.
-  Beneficence: our approach to a transgender individual is non judgmental and understanding. Our main goal is to provide a plan of care for these patients to improve their quality of life and reduce their suffering.

The biggest challenge was to perform sex reassignment surgeries in our devout catholic institution with the presence of religious members on the ethical committee. When the Vatican published its first document rejecting the idea that gender is distinct from biological sex [5], our religious members of the committee gave the authorization to a gender affirming surgery to relieve patients from their social and psychological sufferings.

When Elkak [6] reported that 73% of Lebanese physicians considered homosexuality as a disease and just half of them were willing to offer them medical care, our institution has decided to provide healthcare for every homosexual and transgender patient despite the presence of all these challenges. Legally, providers can present hormone treatment and surgical therapies for trans people who want to undergo gender affirming surgeries [7]. In a country where we criminalize same-sex sexual activity and sex reassignment, and most transwomen are fearful to access health services due to stigma, we decided to present healthcare for everybody without any discrimination. Sexually transmitted disease screening, HIV and HPV screening, hormone treatment, gender affirming surgeries and other services are provided to every Lebanese transgender person in our health clinic. The medical students, the psychologists, the nurses and social workers are trained to provide suitable healthcare to transgender patients. Sufficient knowledge and surgical expertise were enough for a transgender woman to feel safe and in good hands in our clinics. Moreover, the development of a complaints mechanism through which patient can report cases of stigma or discrimination provide security and satisfaction for the patient. For almost 15 years, we successfully have managed to receive many transgender patients who were satisfied with the medical and psychological treatment they received, and we performed six gender affirming surgeries in the presence of all the conflicts.

Finally, this article promotes our practice and conflicts in presenting our healthcare to transgender people. It is true that the approach and the behaviors of providers toward LGBT patients in Lebanon are improving in the last years [8] but spreading awareness and knowledge among providers and changing policies and legislations in our country are essential to protect transgender rights.
